# Detecting Visually Observable Disease Symptoms from Faces

**DOI:** 10.1186/s13637-016-0048-7

**Published:** 2016-08-31

**Authors:** Kuan Wang, Jiebo Luo

**Affiliations:** University of Rochester, Rochester, USA

**Keywords:** Computer vision, Imbalanced dataset, Anomaly detection, Semi-supervised Learning, Classification, Clinical informatics

## Abstract

Recent years have witnessed an increasing interest in the application of machine learning to clinical informatics and healthcare systems. A significant amount of research has been done on healthcare systems based on supervised learning. In this study, we present a generalized solution to detect visually observable symptoms on faces using semi-supervised anomaly detection combined with machine vision algorithms. We rely on the disease-related statistical facts to detect abnormalities and classify them into multiple categories to narrow down the possible medical reasons of detecting. Our method is in contrast with most existing approaches, which are limited by the availability of labeled training data required for supervised learning, and therefore offers the major advantage of flagging any unusual and visually observable symptoms.

## Introduction

Previous works based on machine learning and computer vision [1–4] have shown the commercial potential and the practical value of symptoms detection and classification using computing devices. A generalized algorithm is useful as an independent step before higher-level algorithms like recognition and prediction; the existing recognition algorithms are usually based on assumptions and trained for specific symptoms, therefore the performance and utility are constrained by lacking training data of unusual symptoms.

We propose to adopt semi-supervised anomaly detection combining with computer vision features extracted from normal faces datasets to produce a reliable mechanism of detecting and classifying abnormal symptoms that are visually observable from faces.

This study makes several contributions, including

1. Analyzing and quantifying common facial features which are generally shared among human beings regardless of race, gender and age. The data and results are produced upon applying computer vision algorithms and statistical analysis on faces databases [5]. The actual data in use include more than 8200 frontal face images following gender, age, and race distributions of the adult U.S. population [5].
2. Detecting and categorizing suspected illness features on the testing data by adopting the semi-supervised outliers based on the statistical facts obtained from normal faces dataset. The illness featuring data are collected from UCSD School of Medicine and VA Medical Center [6], The Primary Care Dermatology Society [7], and other multiple online resources [8]. The testing dataset is consisted of 237 pictures of more than 20 diseases (Central CN 7 Palsy, Cervical Adenopathy, Hematoma of the Scalp with Cellulitis, Parotitis, Peripheral CN7 Palsy, Submandibular Abscess, Zoster and Cellulitis, Corneal Ulcer, Cyanosis, Extraocular Muscle Entrapment (Inf Rectus), Horner's Syndrome, Icterus, Muddy Brown Sclera, Periorbital Cellulitis, Periorbital Echymosis, Scleritis, Subconjunctival Hemorrhage and different types of Acnes) which can be reflected as abnormal facial features under a variety of different conditions, and 237 pictures of normal randomly picked from databases [9–13].
3. Unifying multiple symptom-detecting processes for different diseases into one automatic procedure by a relatively simple implementation, such that the recognition of specific diseases can be isolated as an independent module with less assumptions.

**Fig. 1:**
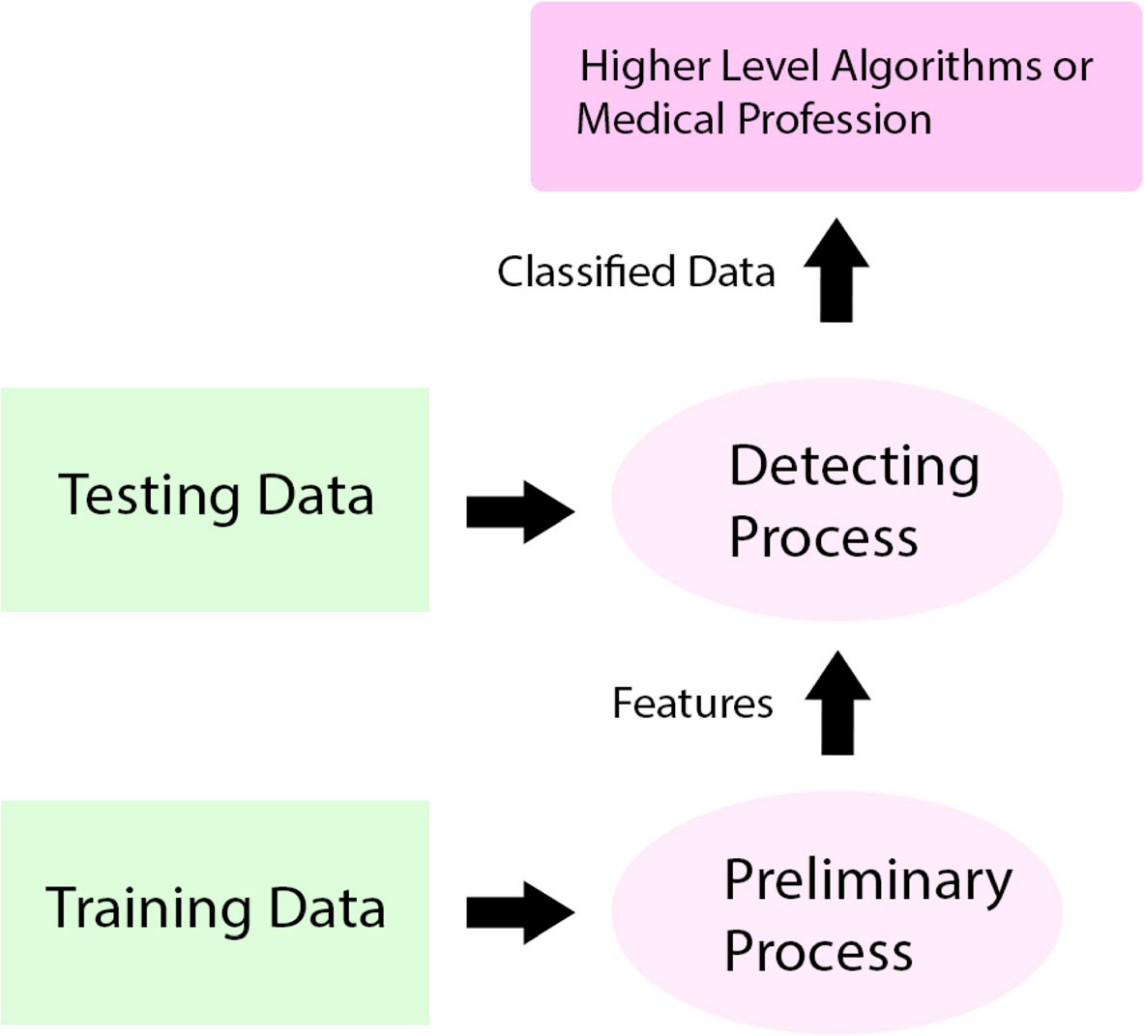
The proposed framework adopting our methods: training data processing and feature extracting are introduced in section 3.1 and 3.2; detecting process running on testing dataset is introduced in section 3.3

Figure 1 displays the workflow of the proposed methods.

The results of this research are expected to be a practical tool for preliminary diagnosis. It could be used as a component of health systems and increase the efficiency of treatment process and makes use of previously unused data. It is important to note that the algorithms introduced in this paper are intended to be a supplementary tool for existing medical assessment and treatment mechanisms, not a replacement.

## Related Works

Early works investigated the utility of systems based on supervised learning, which provide gratifying performance but also require significant feature engineering and high quality training data. Quentin Ferry et al. introduced SVM classifier and PCA to extract phenotypic information from ordinary non-clinical photographs to model human facial dysmorphisms in a multidimensional 'Clinical Face Phenotype Space' [1]; Jane Reilly Delannoy and Tomás E. Ward proposed a computer vision based system for automatically measuring patients’ ability to perform a smile [2], where the degree of facial paralysis can be identified with the aid of Active Appearance Models; Mingjia Liu and Zhenhua Guo introduced an approach to detecting jaundice by investigating skin color with reasonable accuracy [3]; Lilian de Greef et al. introduced a system on mobile phone to monitoring newborn jaundice by analyzing the skin conditions of infants along with color calibration cards [4]. Compared to previous works, our methods focus on detecting and classifying ill faces without assuming specific targeting symptoms by adopting semi-supervised anomaly detection.

## Approaches

For the purpose of detecting multiple symptoms and the future extensibility of our algorithm, we avoided using techniques which are sensitive to specific symptoms only, like the House-Brackman scoring system [2]; instead, we relied on studying the statistical models of general facial features, e.g. color and proportion, as those are likely to be distorted by infections and disorders. Machines perform more sensitive to the eccentricity of statistical data than human beings do, therefore the dependency on special calibrations, like House-Brackman scoring system mentioned above, can be reduced and replaced by those general calibrations with a relatively low cost.

### Data Collecting and Labeling

The training dataset is composed of 8278 pictures of normal frontal face images [5] following gender, age, and race distributions of the adult US population [5]; we further collected 237 pictures of faces with symptoms [6–8] paired with 237 pictures randomly picked from normal face datasets [9–13] as our testing dataset.

#### Training Dataset

The training dataset is composed of 8278 pictures of normal frontal faces [5]. We used active shape models (ASMs) to label this dataset. The algorithm adopted in this study is a reimplementation of Face Alignment by Explicit Shape Regression [14], licensed by MIT. The version of Face Alignment algorithm used in this experiment is trained by the Helen Database [15] with 194 landmarks.

**Fig. 2:**
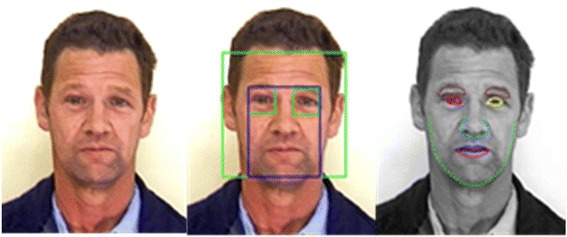
An example of data labeling process by ASMs. Bounding boxes on facial components were applied to increase the precision of the ASMs algorithm

Figure 2 displays an example of data labeling process by ASMs for our training dataset.

#### Testing Dataset

The testing dataset is composed of 237 pictures of ill face [8] expanded from from UCSD School of Medicine and VA Medical Center [6] and The Primary Care Dermatology Society [7] and 237 pictures randomly picked from normal faces datasets [9–13]; 474 pictures in total. 20 diseases are featured in this dataset (Central CN 7 Palsy, Cervical Adenopathy, Hematoma of the Scalp with Cellulitis, Parotitis, Peripheral CN7 Palsy, Submandibular Abscess, Zoster and Cellulitis, CN3 Palsy, Corneal Ulcer, Cyanosis, Extraoccular Muscle Entrapment (Inf Rectus), Horner's Syndrome, Icterus, Muddy Brown Sclera, Periorbital Cellulitis, Periorbital Echymosis, Scleritis, Subconjunctival Hemorrhage and different types of Acnes).

Source URLs for our collected testing dataset [8] were converted to shortened versions for the purposes of publication using TinyUrl (http://tinyurl.com/). The links provided are expected to decay with time and should only be considered exemplars of database composition.

We paired 237 pictures of face with symptoms with equal amount of normal faces data because we had no information about the prior probabilities of various diseases. On the other hand, it is common to evaluate the performance of a system by assuming an equal prior when the actual prior is highly skewed because a trivial classifier that always predicts the popular class will seemingly do extremely well.

The 237 pictures of face with symptoms in the testing dataset were hand labeled. Most of those images were collected along with mosaics or clipped to protect personal privacy; therefore, ASMs were not applicable to them. Applying ASM algorithm on images with those unpredictable conditions is another different challenging problem. Since it is not directly related to the challenge addressed in this paper, we decided to hand label this testing dataset for simplifying purpose. We plan on addressing this problem in future work.

Hand labeling mostly followed the same criteria of ASMs algorithm used in training dataset; we did not label the features were not observable, for example, eyes covered by mosaic; we labeled the skin area only if no common facial feature in the image.

**Fig. 3:**
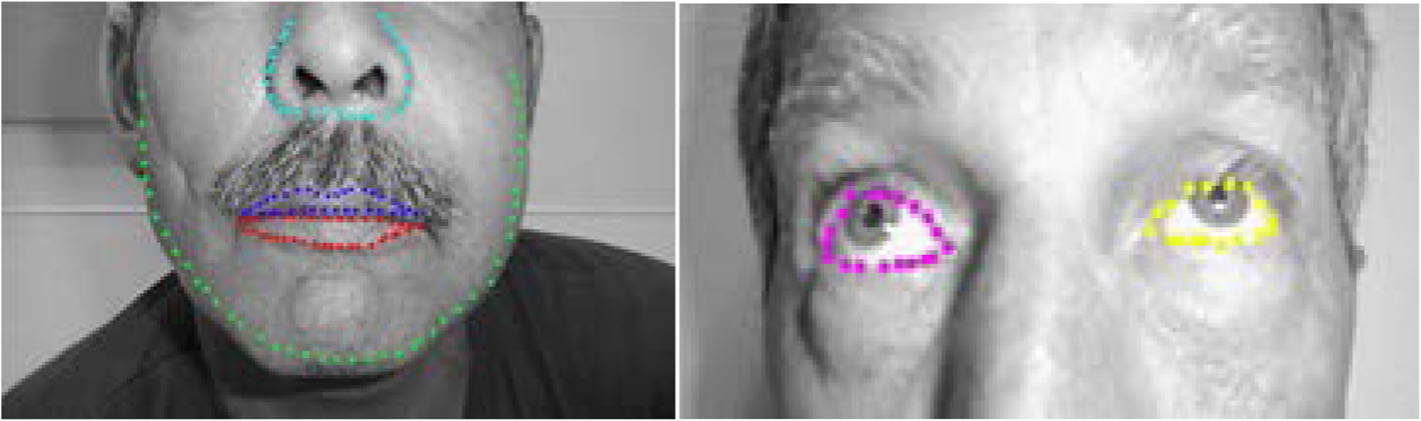
Two examples of hand labeled data for faces with symptoms. Hand labeling followed the same criteria of the ASMs algorithms

Figure 3 displays two examples of hand labeled data in our testing data set.

### Feature Extraction

The labels we made in the pictures of the training dataset and the testing dataset suggested the polygons that bounded all related pixels for certain face components, for example, left eye and lips. For each set of labels, we obtained its binary imaging to represent its corresponding facial component.

Because of the limits of the ASMs with 194 landmarks, some labels overlapped with each other, therefore one pixel could be incorrectly presented in more than one binary feature; for example, the upper lip might share regions with the lower lip. The overlapping pixels usually represent neither lips, but the teeth and tongue on a smiling face, which are not the region of interest in our experiments. To avoid including errors, we further sanitized the features by removing those overlapping pixels.

We transformed the original picture from the RGB color space into the CIELAB color space. The A channel and B channel of CIELAB color space allows an approximately linear scale describing the redness and yellowness of the features to flag the potential symptoms on faces. Combining with extracted binary features, we could have a better understanding of the size, color, proportion and even relative position of those face components.

**Fig. 4:**
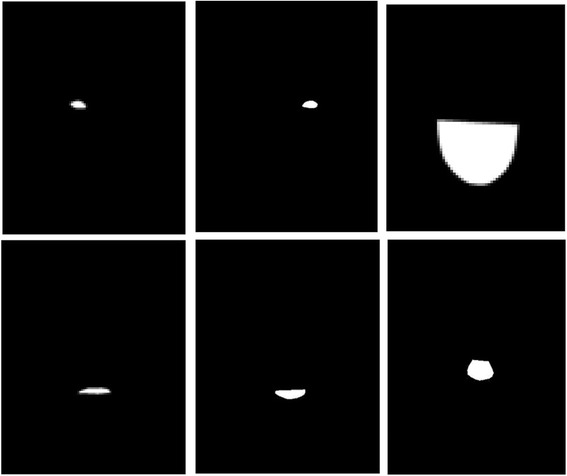
A set of binary features on a face corresponding to left eye, right eye, face contour, upper lip, lower lip and nose

For one picture of a face, we extracted six possible binary features: face/skin, upper lip, lower lip, nose, left eye and right eye (Fig. 4). The extracted binary features obtained from labeled data were used in future steps to generate variants for anomaly detection algorithm.

### Anomaly Detection

Because the prior probabilities of diseases were unknown, we instead assumed Guassian distribution on the features of our normal face data in this preliminary study. We defined an outlier as one observation containing at least one variant that appearing to deviate markedly from the obtained mean value of the samples in the training dataset.

#### Variants Selection and Extraction

Table 1 illustrates the variants we used in the outlier detection and their statistical summarization obtained. For abbreviation, α represents the aggregate value of the CIELAB alpha channel (red-green channel) of the feature; β represents the aggregate value of the CIELAB beta channel (yellow-blue channel) of the interested feature; Σ represents the total count of all the pixels belonging to the feature; H is the process of applying the well-known Hough Transform on the CIELAB feature of the skin area, and then further applying a counting function to count how many circular structures we found; the mechanism is based on Size Invariant Circle Detection [16].

**Table 1:** Variants for the outlier detection algorithm, with their mean values and corresponding standard deviation

	Variant	μ	δ
1	α(Eye)/Σ(Eye)	138.426	12.412
2	β(Eye)/Σ(Eye)	138.214	13.345
3	α(Lip)/Σ(Lip)	150.725	9.752
4	Σ(LFace)/ Σ(RFace)	0.962	0.186
5	Σ(LEye)/ Σ(REye)	0.958	0.071
6	H(Face)	2.233	3.141

For Variant 4, the middle line of a face was defined as the line passing through the middle label of the nose and the middle label of the face contour because the labels of ASM algorithm were indexed.

For Variant 6, the K-Means Clustering algorithm was applied on the CIELAB feature before applying size invariant circle detection in our experiments. The clustered features of symptom featuring faces are usually rigid; we further applied Hough Transform on the clusters to find potential circular structures.

**Fig. 5:**
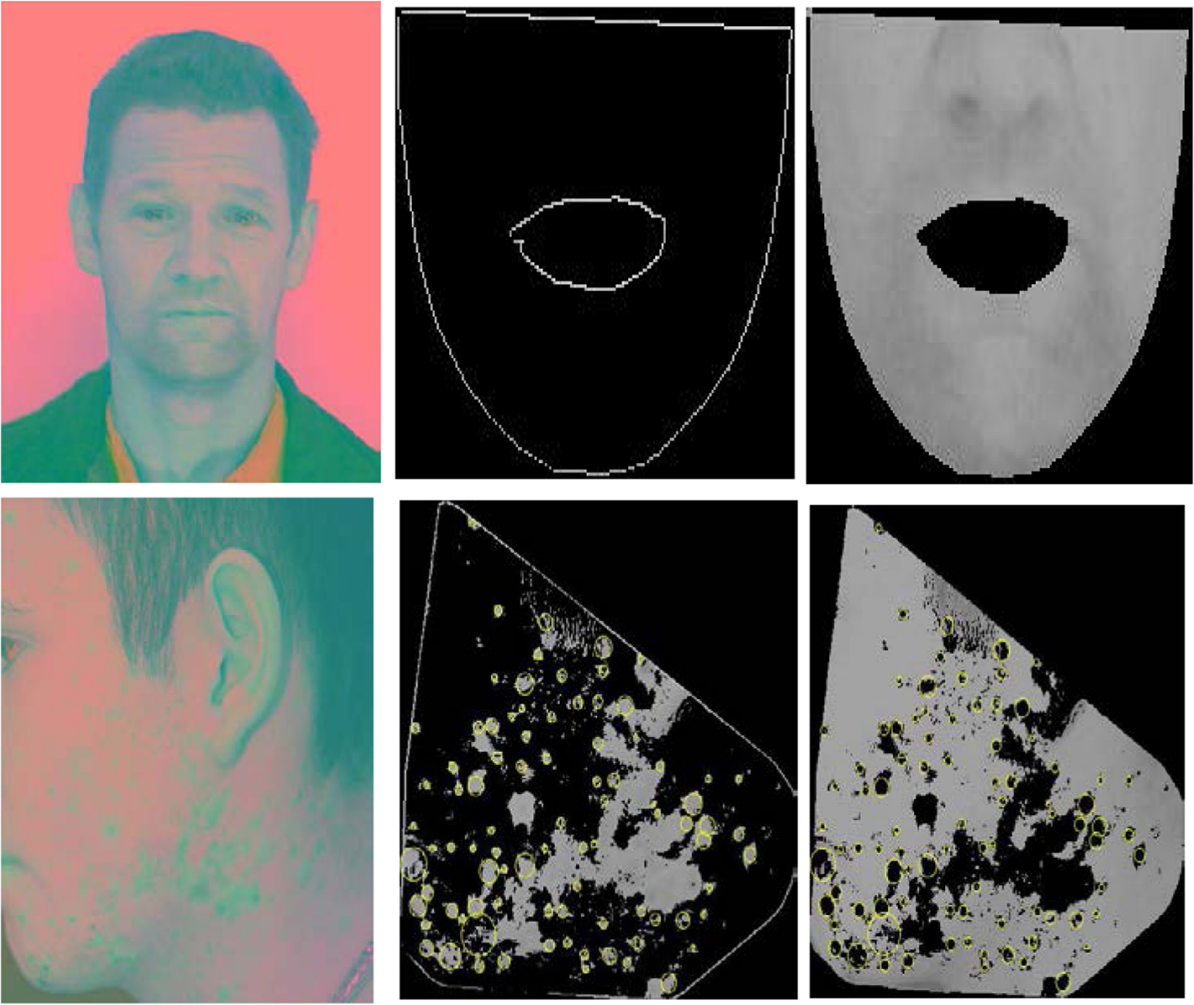
Two sets of features for Variant 6. 1st row illustrates the CIELAB features and their clusters of a normal face; 2nd row illustrates the CIELAB features and their clusters of a face with acne. Yellow circles are the results of applying Size Invariant circle detection. For above examples, 0 circle was found for the normal face; 119 circles were found for the acne featuring face

Figure 5 displays a comparison of exploring Variant 6 on a normal example and an abnormal example.

In Table 1, Variants 1–3 reflect the color properties (average color); Variants 4–5 reflect the proportion properties; Variant 6 reflect any other special features that one normal face should not contain.

The values of those variants listed in Table 1 can be easily computed by investigating binary features and the corresponding CIELAB feature. We further summarized the mean values (μ) and standard deviations (δ) of the data in training dataset. An outlier is hence defined as a variant whose value is not in μ ± t × δ, where t is the multipler we used to tighten the degree of normality. We applied the threshold μ ± t × δ on our observations with assumed distribution function and eventually divided the testing dataset into flagged group and unflagged group with respect to different t values.

For those data with no certain binary features because of the data quality issue, some variants were not applicable, e.g., *Variant 1* (the redness of eye) could not be applicable because no corresponding binary feature of eyes was available for this picture. In addition, color related variants require colored images; proportion related variants require frontal face images.

## Results

In this study, we picked the threshold *t* from *t* = 0.0 to *t* = 3.0, with the interval of 0.1, 31 sets of experiments in total. The statistical results are shown in Table 2.

**Table 2:** Statistical results collected by choosing thresholds from *t* = 0.0 to 3.0

*t* =	TP	FP	Precision	Recall	Accuracy	F-1
0.0	237	237	0.500	1.000	0.500	0.667
0.1	237	237	0.500	1.000	0.500	0.667
0.2	236	237	0.499	0.996	0.498	0.665
0.3	232	235	0.497	0.979	0.494	0.659
0.4	231	229	0.502	0.975	0.504	0.663
0.5	229	221	0.509	0.966	0.517	0.667
0.6	222	186	0.544	0.937	0.576	0.688
0.7	218	175	0.555	0.920	0.591	0.692
0.8	216	154	0.584	0.911	0.631	0.712
0.9	212	125	0.629	0.895	0.684	0.739
1.0	209	110	0.655	0.882	0.709	0.752
1.1	207	97	0.681	0.873	0.732	0.765
1.2	200	77	0.722	0.844	0.759	0.778
1.3	196	71	0.734	0.827	0.764	0.778
1.4	191	65	0.746	0.806	0.766	0.775
1.5	186	58	0.762	0.785	0.770	0.773
1.6	183	40	0.821	0.772	0.802	0.796
1.7	176	35	0.834	0.743	0.797	0.786
1.8	171	33	0.838	0.722	0.791	0.776
1.9	163	25	0.867	0.688	0.791	0.767
2.0	160	22	0.879	0.675	0.791	0.764
2.1	157	19	0.892	0.662	0.791	0.760
2.2	155	18	0.896	0.654	0.789	0.756
2.3	151	17	0.899	0.637	0.783	0.746
2.4	145	14	0.912	0.612	0.776	0.732
2.5	144	13	0.917	0.608	0.776	0.731
2.6	141	10	0.934	0.595	0.776	0.727
2.7	139	9	0.939	0.586	0.774	0.722
2.8	136	5	0.965	0.574	0.776	0.720
2.9	134	5	0.964	0.565	0.772	0.713
3.0	133	4	0.971	0.561	0.772	0.711

We collected 60 pictures of 20 different diseases from UCSD School of Medicine and VA Medical Center [6] and The Primary Care Dermatology Society [7] as our starting point, and then expanded this dataset by collecting the images with the same descriptions from other online resources. We eventually obtained 237 pictures of faces with symptoms [8]. In this way the professional suggestions and symptom descriptions [6, 7] are also applicable to this expanded dataset.

**Fig. 6:**
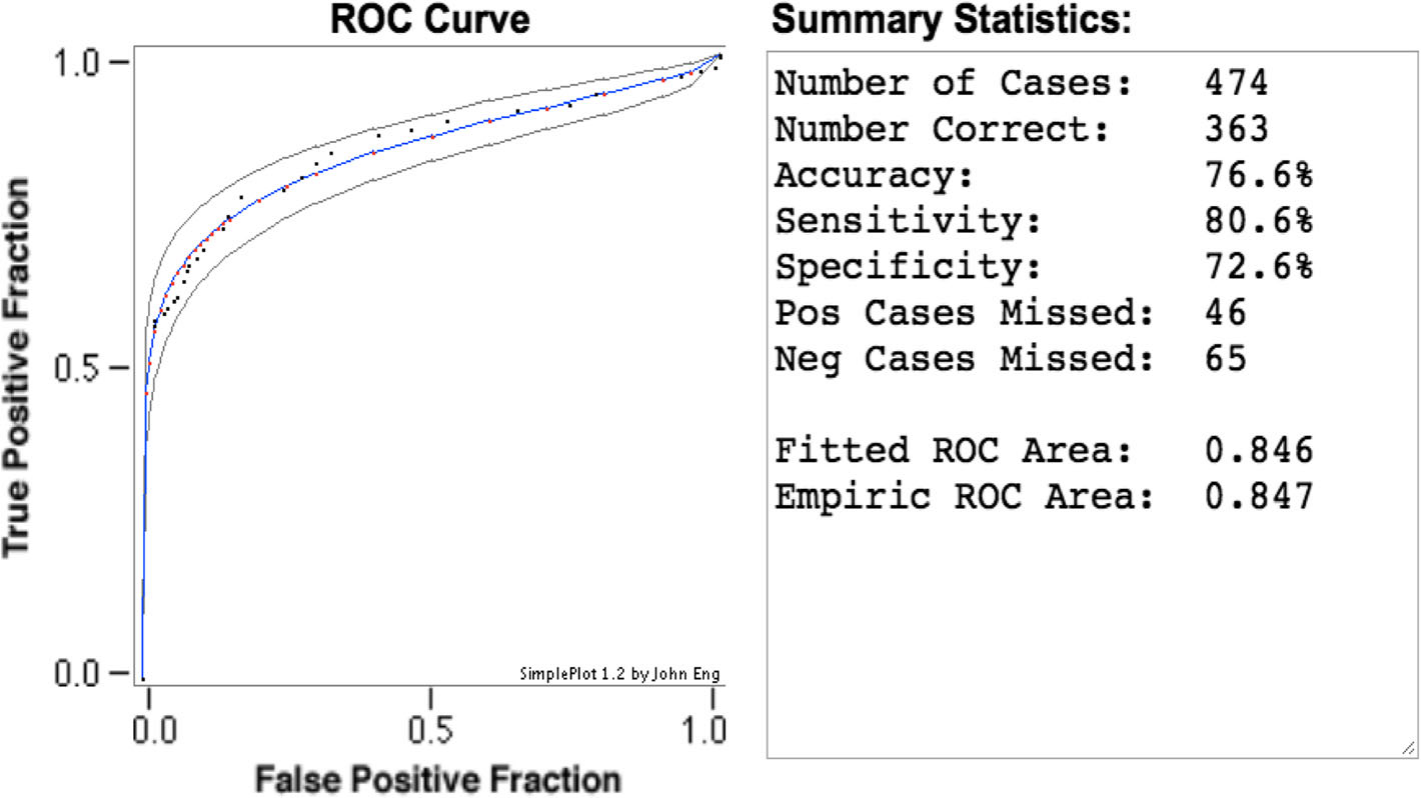
The ROC curve computed by statistical data in Table 2, and its statistical summary

Figure 6 displays the ROC curve computed with the 31 sets of experiments displayed in Table 2, using the maximum likelihood fit of a binormal model [17]. The fitted ROC Area (AUC) is 0.846; the Area under curve (AUC) evaluates the overall performance of the algorithm.

**Fig. 7:**
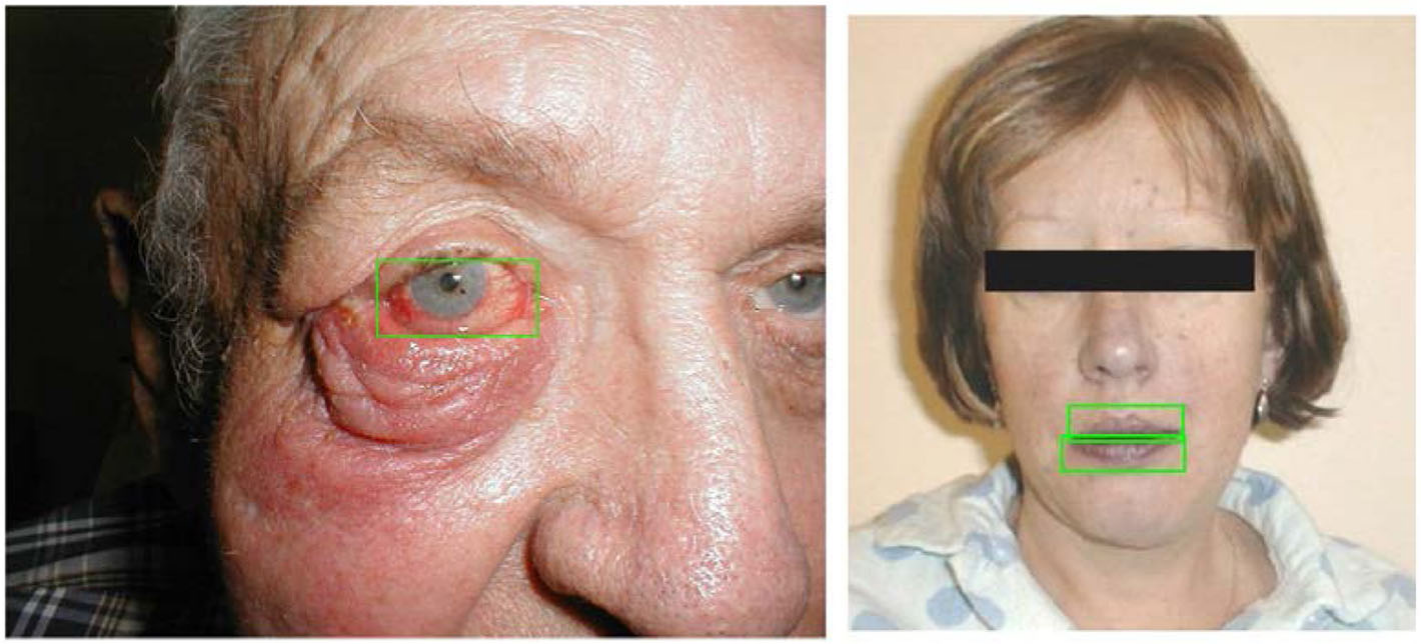
True positive examples flagged by the outlier detection. Left: Periorbital Cellulitis; right: Cyanosis

**Fig. 8:**
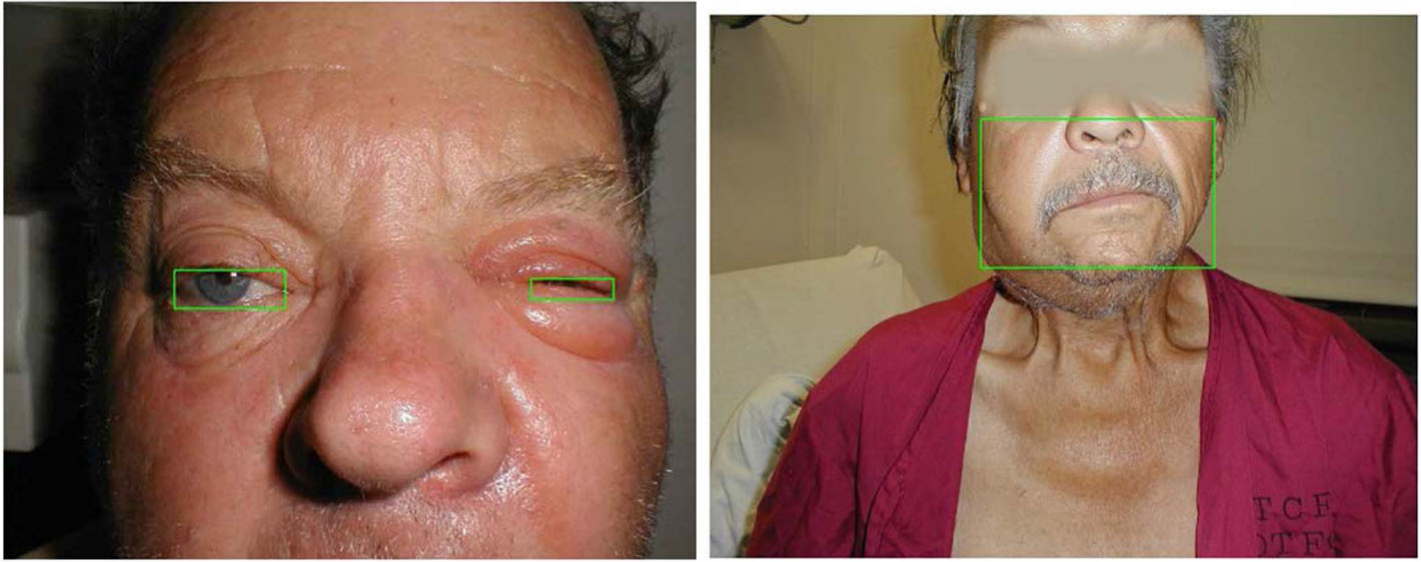
True positive examples flagged by the outlier detection. Left: Periorbital Cellulitis; right: Cervical Adenopathy

Figures 7 and 8 display some examples of the detection of the true positive data.

Figure 7 displays two outliers at t = 1.0 captured because of color information; the left picture was flagged as an outlier by Variant 1 (i.e., redness of eyes, value = 168); the right picture was flagged as an outlier by Variant 3 (i.e., lips color, value = 139).

Figure 8 displays two outliers at *t* = 1.0 captured because of proportion information; the left picture was flagged as an outlier by Variant 5 (i.e., proportion of eyes, value = 3.03); the right picture was flagged as an outlier by Variant 4 (i.e., proportion of face, value = 1.71).

We also recorded the variant flagged each outlier and the its value; we compared these factors with the ground truth; the flagged cases were counted as true positive reports reflected in Table 2 only if the variants matched the ground truth; we further classified these true positive reports into multiple categories by the reporting variants. The results were displayed in Table 3.

**Table 3:** Six categories corresponding to their flagging reasons

	Flagging Reason	Suspected Symptoms
Category 1	Variant 1 > μ + *t* × δ	Scleritis, Subconjunctival Hemorrhage, Corneal Ulcer, Extraocular Muscle Entrapment (Inf Rectus), Muddy Brown Sclera, Periorbital Cellulitis, Periorbital Echymosis
Category 2	Variant 2 > μ + *t* × δ	Icterus
Category 3	Variant 3 < μ + *t* × δ	Cyanosis
Category 4	Variant 4 > μ + *t* × δ or Variant 4 > μ - *t* × δ	Central CN 7 Palsy, Cervical Adenopathy, Parotitis, Peripheral CN7 Palsy, Submandibular Abscess
Category 5	Variant 5 > μ + *t* × δ or Variant 5 > μ - *t* × δ	Central CN 7 Palsy, Peripheral CN7 Palsy, Extraocular Muscle Entrapment (Inf Rectus), Horner’s Syndrome, Periorbital Cellulitis, Periorbital Echymosis
Category 6	Variant 6 > μ + *t* × δ	Acnes, Hematoma of the Scalp with Cellulitis, Zoster and Cellulitis

Each flagged outlier was classified into one of the six categories according to its reporting variant. Although most of the categories contain more than one suspected symptoms, the classified category helps to narrow down the possible medical reasons of the anomaly detection

## Conclusions and future Works

For the purpose of our study, a dataset containing a wide range of diversities of symptoms with roughly equal amount of each is required for testing; similar data scarcity challenge is also faced by many other studies on image recognition-based diagnosis [1–4]. We address this problem by using semi-supervised anomaly detection which produced promising results. Given the diversity, imbalance, and noise in the dataset, as well as a simple methodology, the statistical results we achieved in this study confirm the promise of our approach and future possibilities.

However, semi-supervised learning also restrained the performance. Algoritms for medical usages often require high recall with relatively high precision, which is still beyond the overall summary statistics of our current methods. There are other semi-supervised anomaly detecting mechanisms could be used [18]. We investigated Gaussian Model-Based detecting mechanism in our preliminary study; applying other semi-supervised anomaly detecting models on our variants should result in similar performance. We plan to improve the performance of our algorithm in future work.

The better results could be obtained by combining multiple variants; as implied in Table 3, some diseases have symptoms reflected by multiple variants. However, it would be nontrivial to learn such correlation for the number of variants without supervision. Given that our proposed system is motivated by avoiding using expensive supervised learning, exploiting the correlation between multiple variants is out of the scope of this study.

Our algorithm can be integrated into a multi-cue diagnosis system, i.e. Visual Clinial Decision Support System (CDSS), to help a clinician make a final, reliable diagnosis decision combining with temperature, lab test and other observations. We have initiated some collaborations on automated skin lesion characterization in the context of CDSS; we plan to deploy our methods to industrial pipelines to validate and improve our methods. The anomality detecting mechanism introduced in this study can also be extended to assist other health related research like detecting and recognizing psycho-behavioral signals [19]. In addition, while our study focuses on the faces, the algorithm itself is readily extended to body and limbs.
